# Hepatitis E Virus in the Wild Boar Population: What Is the Real Zoonotic Risk in Portugal?

**DOI:** 10.3390/tropicalmed8090433

**Published:** 2023-08-31

**Authors:** Ana Carolina Abrantes, Sérgio Santos-Silva, João Mesquita, Madalena Vieira-Pinto

**Affiliations:** 1 Animal and Veterinary Research Centre (CECAV), Trás-os-Montes e Alto Douro University (UTAD), 5000-801 Vila Real, Portugal; 2 Abel Salazar Institute of Biomedical Sciences (ICBAS), University of Porto, 4050-313 Porto, Portugal; 3 Epidemiology Research Unit (EPIunit), Institute of Public Health, University of Porto, 4050-600 Porto, Portugal; 4 Laboratory for Integrative and Translational Research in Population Health (ITR), 4050-600 Porto, Portugal; 5 Department of Veterinary Sciences, Trás-os-Montes e Alto Douro University (UTAD), 5000-801 Vila Real, Portugal; 6 AL4AnimalS-Associate Laboratory for Animal and Veterinary Sciences, Portugal

**Keywords:** emerging infectious disease, foodborne disease, molecular diagnostic, serosurvey

## Abstract

Hepatitis E virus (HEV) is an important zoonosis in wild boar. Reported zoonotic cases are mainly associated with the consumption of raw/undercooked meat and/or liver. This study aims to determine the occurrence of HEV in the Portuguese wild boar population. During the hunting season 2021/2022, 123-matched samples (liver, faeces, and blood) were collected from hunted wild boars throughout Portugal. An RT-PCR assay tested liver and faeces samples to detect HEV-RNA. From blood samples, an ELISA test was performed. Only one liver sample was positive for HEV (0,8%) and one other from faeces. A total of 34 sera were seropositive (26.7%). At the same time, in a survey of 106 hunters, 21 consumed/ate the liver of wild boars (19.8%). Only three recognised the possibility of consuming it undercooked. Contrary to previous studies in Portugal, the prevalence of HEV in liver and faeces is low, but the seropositivity is higher. But, when analyzing in detail, it could be observed that an HEV hotspot exists in the southeast of central Portugal and that it is a zoonotic risk for hunters of this region. The data of this study reinforce the importance of including HEV in surveillance programs for wildlife diseases to expand the potential zoonotic information.

## 1. Introduction

One of the most studied viruses circulating through the animal population with zoonotic potential is the Hepatitis E virus (HEV) [1,2]. Hepatitis E is a liver disease caused by the Hepatitis E virus (HEV), a non-enveloped positive-sense single-stranded RNA virus classified as *Paslahepevirus balayani*, which includes eight major genotypes (HEV genotype 1 to genotype 8). HEV-1 and HEV-2 cause infections in humans in developing countries, where they are transmitted primarily by the faec–oral route of consuming contaminated water or food [3,4,5,6]. While HEV-1 and HEV-2 only affect human beings, HEV-3 and HEV-4 are zoonotic, infecting several animal species beyond humans [7,8]. In humans, this virus causes a usually self-limiting liver disease with a mortality rate generally below 1%, although 15–25% mortality rates have been recorded in pregnant women. Chronic infections can also occur [3].

EFSA data report that autochthonous cases have increased over recent years in Europe and are mainly associated with HEV-3 infections. Pigs and wild boars are considered the main reservoirs [9].

In fact, several authors have already emphasized the importance of wild boar as a vector of many zoonotic pathogens [2,10], especially foodborne pathogens [11], as in the case of HEV. This topic is of increased concern due to the recent growing consumption of wild boar meat. This meat has huge natural properties and a small ecological footprint [12]. Under this context, wild boar has been identified in different countries as a risk factor for autochthonous HEV infections. Being reported in several European countries as one of the microorganisms with the most significant importance in terms of the potential zoonotic risk, it presents due to its epidemiological context, hosts, interactions with humans, and risk factors present in these habitats/environments [2,13]. In this case, human infection can occur through direct contact with infected animals or by the consumption of raw or undercooked wild boar meat, the liver being the edible product with the higher risk. Liver is considered the primary target site of HEV replication in swine and an essential organ in the pathogenesis of the disease [14]. It should also be noted that although liver is the preferred site for viral replication, extrahepatic replication and viremia processes can lead to viral circulation in blood and muscle [15,16]. However, in the wild boar, periodic viral shedding occurs through the faeces [15,17].

Aprea et al. (2018) [3] described several foodborne transmissions of HEV-3 and HEV-4 in industrialized countries. Most were linked to the ingestion of uncooked wild boar meat and raw pork liver, like the one described by Rivero-Juarez et al. (2017) that demonstrated by phylogenetic analysis of zoonotic transmission of HEV by wild boar meat consumption in Spain [3,18]. Also, studies developed by Widen et al. (2011) in Sweden have shown a relation between human HEV strains and wild boars [19]. According to Aprea et al. (2018), this disease is recognized as an emerging zoonosis, with a marked circulation in the European wild boar populations, and the dispersal of HEV raises public health concerns, mainly due to self-consumption of wild boar meat without authorities’ control [2,18,20,21]. The same occurs in Portugal, a country with similar epidemiological scenarios to Italy and Spain. By far, studies of HEV infection in wild boar are scarce [2], and the zoonotic risk potential has not been analysed until now.

Hence, hunters are a high-risk zoonotic population in this context due to hunting traditions, to Portuguese hunters’ consumption habits, and also to self-consumption of wild boar meat and liver. It is, therefore, essential to assess the risk to which they are exposed and whether their consumption and hygiene practices increase the risk of acquiring HEV during the evisceration, manipulation, and consumption of wild boar [16,22].

This study aims to determine the occurrence of HEV in the wild boar population in Portugal and discuss the potential zoonotic risk of this in a target population, such as Portuguese hunters.

## 2. Materials and Methods

### 2.1. Study Area and Sampling

**Figure 1:**
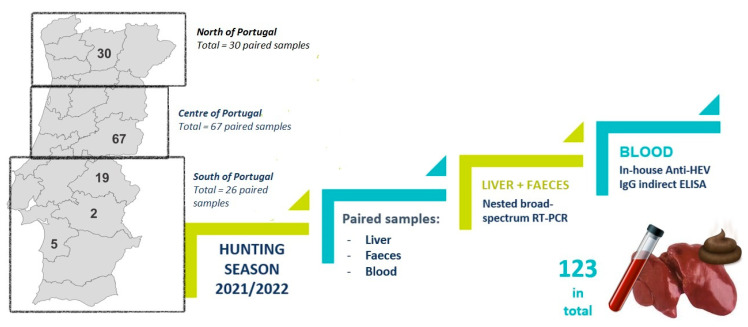
Geographical distribution of wild boar samples (liver, faeces, and blood) in Portugal and schematic sample collection and laboratory procedures.

During the hunting season 2021/2022 (October 2021 to February 2022), 123-matched samples (liver and blood) were collected post-harvest from wild boars from different areas throughout Portugal [30 from the north of Portugal, 67 from the center (mainly southeast), and 26 from the south] (Figure 1). All samples were collected from wild boars legally hunted. This study did not involve the deliberate killing of animals.

Within a short time after death, during the evisceration in the collection point after each driven hunt, matched samples were collected: a liver sample (~75 g), faecal samples (~25 g) from the posterior part of the large intestine, and blood (~2 mL) taken from the animal’s chest cavity. All samples were kept at 4 °C and transported to the lab within 12 h. Faecal and liver samples were stored at −20 °C until DNA/RNA extraction. After centrifuging the blood samples, the serum collected was stored in the same conditions until tested. All serum samples were tested for anti-HEV antibodies using an immunological assay (ELISA test), and all faecal and liver samples were tested for HEV RNA using molecular assays (nest-PCR).

Each sample has associated data: location hunted (County and District), sex (male/female), and age (adult/subadult).

### 2.2. Molecular Detection of HEV in Liver and Faeces

In the laboratory, 123 matched samples were tested for HEV using PCR assays after the extraction process of the viral RNA existent in the livers and faeces.

Liver samples were homogenised after storage at −180 °C, prepared in phosphate-buffered saline (PBS) pH 7.2, and centrifuged for 5 min at 8000× *g*. According to Zhao and Li (2021) [23], after liver homogenisation, the mixtures were clarified using centrifugation at 10,000 rpm for 20 min. The supernatant was transferred to a sterile Eppendorf^®^ tube and incubated at 55 °C for 10 min with 1 µL proteinase K solution. After protein digestion, the samples were centrifuged at 10,000 rpm for 20 min. Faecal suspensions were prepared in phosphate-buffered saline (PBS) pH 7.2 and centrifuged for 5 min at 8000× *g*, as well as the liver samples. RNA was extracted in both samples (liver and faeces) and purified using the QIAamp Cador Pathogen Mini Kit^®^ (Qiagen, Hilden, Germany), according to the manufacturer’s instructions using 200 µL of the clarified supernatants in the QIAcube automated platform (Qiagen^®^, Hilden, Germany). Eluted RNA was stored at −80 °C with RNase-free water.

According to Johne et al. (2010) [24], a nested broad-spectrum RT-PCR assay was used for the molecular detection of HEV in the matched samples. PCR reaction was performed on a CFX Connect Real-Time PCR Detection System (Bio-Rad; Hercules, CA, USA) and a T100 thermocycler (Bio-Rad), with reaction mixtures as Fast qPCR Mastermix (Probe) (GriSP^®^, Porto, Portugal), Fast PCR Mastermix (GriSP^®^, Oporto, Portugal), and 2× Xpert Fast Hotsart Mastemix (GriSP^®^). Amplified RNA fragments were identified using electrophoresis of PCR amplification products at 100 V for 40 min on 1.5% agarose gels stained with Xpert Green Safe DNA gel dye (GriSP^®^, Oporto, Portugal). UV light was irradiated to confirm the results.

Using GRS PCR and a Gel Band Purification Kit (GriSP^®^, Oporto, Portugal), amplicons that appeared to be positive were purified, and bidirectional sequencing was carried out. With the help of the BioEdit Sequence Alignment Editor v7.1.9 software package, version 2.1, sequences were aligned and compared to sequences found in the NCBI (GenBank) nucleotide database.

The sequences reported in this study and additional representative sequences were obtained from GenBank and MEGA version X software [25] and the Interactive Tree Of Life (iTOL) platform [26] used for the phylogenetic analysis. This study was inferred using the maximum-likelihood (ML) method [25,26,27], and the Tamura–Nei model was used to calculate the ML bootstrap values using 1000 replicates [27]. The most efficient replacement model was the Tamura–Nei model by MEGA version X [25].

### 2.3. Serologic Detection of HEV

Blood samples collected in laboratory were centrifuged and stored until used at −20 °C. With the serum obtained, an in-house anti-HEV antibody using a commercial double-antigen multi-species sandwich HEV ELISA kit (MP Diagnostics, Illkirch, France) was performed according to the manufacturer’s instructions.

### 2.4. Hunters’ Survey

In parallel, a survey of 106 hunters from all over Portugal was carried out, where the majority of hunters eviscerated wild boar carcasses for private consumption. The survey was distributed randomly during the driven hunts, and all hunters filled out an informed consent. In the survey on “game meat self-consumption and good hygiene practices”, three questions related to risk factors for zoonotic exposure to HEV were asked: two questions about liver self-consumption “Do you usually consume the liver of the carcasses that you eviscerate and prepare?” and “If so, is there any possibility of consuming the liver undercooked?”, with one other question about one tradition of Portuguese hunting activities named hunter’s baptism, “Have you ever seen a hunter’s baptism, the liver being rubbed on the hunter’s face?”

### 2.5. Statstistical Analysis

For the statistical analysis and to calculate the -association between results and risk factors (calculated in other scientific publications as risk factors for the HEV infection in wild boar [4]), such as age, sex, and geographic localization, the following tests were used: Fisher’s exact Test and Odds ratio (OD). A probability value (*p*-value) < 0.05 was considered statistically significant. Both values were calculated using the EpiTool version 0.5–6 statistical analysis software.

## 3. Results

### 3.1. Molecular Detection of HEV in Liver and Faeces

**Figure 2:**
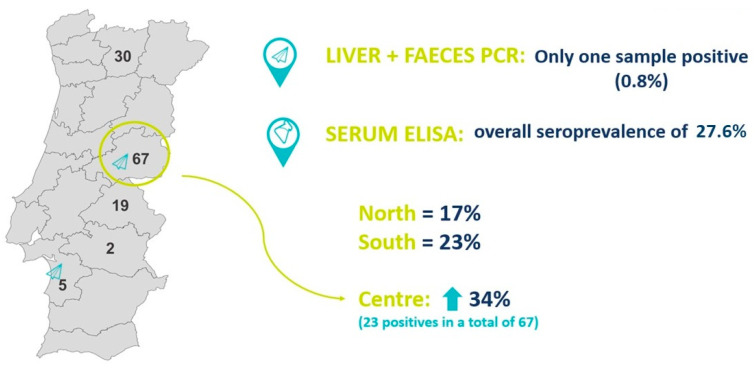
Geographical representation of HEV occurrence (molecular detection in liver and faeces and seroprevalence) results in the Portuguese wild boar population.

The liver is considered the gold-standard sample, but in this study, only one sample was positive for HEV (0.8%). The positive liver was sampled from wild boar hunted in Central Portugal and was an adult female wild boar (Figure 2).

The overall prevalence of HEV in the faeces is the same as in the liver testing, only 0.8% (1 positive sample in a total of 123). In this case, the positive sample came from a wild boar hunted in southern Portugal and was an adult male wild boar (Figure 2).

**Figure 3:**
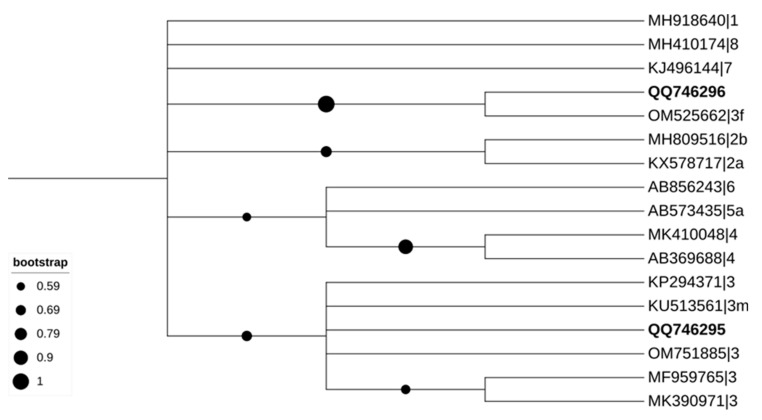
Phylogenetic analysis of HEV obtained in wild boars. Tree derived from 17 nucleotides HEV sequences at the RNA-dependent RNA-polymerase, including sequences from this study (HEV-3 and its accession number are in bold) and 15 strains of various genotypes obtained from GenBank (HEV-1 to HEV-8) (no bold or shading and identified with the accession number as well as its genotype and subgenotype). Tree inferred using the MEGA X maximum likelihood method (Tamura–Nei model) and the Interactive Tree of Life (iTOL).

Thus, two samples were positive for HEV. Amplicons from the positive liver generated a sequence sharing 94.55% identity with a wild boar isolate from Portugal (OM751885), and the positive faecal sample shared 97.22% identity with a wild boar isolate from Spain (OM525662). Both HEV sequences belonged to genotype 3 (Figure 3). Our HEV isolates were deposited in the GenBank with accession numbers: OQ746295 (HEV in liver sample) and OQ746296 (HEV in faeces sample).

### 3.2. Serologic Detection of HEV

Thirty-four positive (n = 34) sera samples were processed in the laboratory, and an ELISA test was performed, with an overall seroprevalence of 27.6%. Notably, in the north and south of Portugal, the seroprevalence is similar (17% in the north and 23% in the south), but in the central area, the seroprevalence increased to 34% (23 positives in a total of 67). Statistical analysis did not reveal a significant association between any region of Portugal (north vs. centre vs. south) and the HEV infection in the sampled wild boar population (Figure 2).

Regarding sex and age data of the sampled animals, the statistical analysis revealed a significant association between age (adult vs. subadult) and HEV seropositivity (*p* < 0.05). The probability of HEV seropositivity in an adult wild boar was 4.13 times higher than in a subadult animal (OR = 4.13). About the sampled animals’ sex (male vs. female), no significant statistical association was found.

### 3.3. Results of the Hunters’ Survey

Of the 106 hunters from all over Portugal (Table 1), 81 have never consumed the liver of the wild boars they hunt (76.4%), but 21 answered sometimes (19.8%), and 4 hunters answered always (3.8%). Of those who consumed the liver, only three recognised the possibility that the liver is occasionally undercooked. As for the question related to the baptism of hunters and the traditional practice of rubbing raw liver on the face, 52 answered that they had never seen this practice happen (49%), but 50 answered sometimes (47.2%) and the remaining 4 always (3.8%).

## 4. Discussion

HEV prevalence in wild boar across Europe has been variable in the last 15 years, dependent on the sample type and typology of the diagnostic method. Reported serologic and molecular HEV prevalence in wild boar has fluctuated widely over the last few years [2].

Data on the wild boar meat (including meat and edible viscera, such as liver) distribution of HEV RNA were detected using RT-PCR and ranged between 1.9% and 52.2% [4,9,28,29,30,31]. Therefore, higher values of HEV RNA (ranging from 10.2% to 25.6%) were detected in wild boar livers. All European studies with the highest values of HEV detection in livers were observed in different regions from Italy, as shown in surveys by Aprea et al. (2018), De Sabato et al. (2020) and Di Pasquale et al. (2019) [3,8,9], and with the absolute highest value (52.2%), as observed by De Sabato et al. in 2018 [4,9,32]. Apart from Italy, other countries have reported surveys with above-average HEV values in livers, such as in Romania by Porea et al. (2017) [13], in Portugal by Mesquita et al. (2016) [30], and in Lithuania in 2018 [31]. With the lowest prevalence values reported in Europe in the last years, other surveys are presented, e.g., 2.45% in livers by Pierini et al. in 2021 in Italy [33] and 5.8% by Lhomme et al. in 2015 in France [34], and consequently others in Spain [14] and Belgium [35]. Contrary to these surveys, in our study, the value presented is much lower, that is, only 0.8%.

To our knowledge, this is the second report documenting the occurrence and molecular analysis of HEV in wild boar in Portugal. But, while Mesquita et al. (2016) described 25% of HEV in their liver samples [30], in our study, the presented prevalence is lower. It ranks as the only one in Europe in the last 20 years with the lowest prevalence (0.8%) of molecular detection using RT-PCR in wild boar livers.

Some of the studies presented reported other positive samples using molecular diagnostic tests besides the liver, like muscle and blood. In most of these samples, the percentage value was lower than that one observed in the liver. Thus, when we approach the panorama of HEV detection using molecular analysis in faeces, our study is already more in line with what has been presented in Europe in recent years. Giving an overall prevalence of 0.8% (1 positive sample in 123 faeces analysed) is similar to the 2.8% (3 positives in 144 faeces samples) reported by Santos-Silva et al. in Portugal with similar sample collection dates [36]. Other studies have reported a prevalence of HEV in faeces from 9.4% in Italy to 20% in Spain over the years [30,37,38].

According to several authors, various factors can be suggested to be involved in prevalence differences of HEV, such as the animals’ stage of infection at the moment of sampling, the age of the examined animals, the population density and the frequency of contact with other wild or domestic receptive species, the ecological differences of wild boar populations, and the phenotypic character [4,13]. Also, prevalence values can vary according to the sample type [4,13] and diagnostic methods [39]. This may explain why the two positive samples (one in the liver and one in the faeces) are not from the same animal in our study and distinct areas of sample collection. Since we would expect that if faeces were positive, so would the liver and vice versa. It is known that the liver is the gold-standard site of viral replication, where the virus should accumulate. Many studies have shown that the median genome copy number in wild boar liver values was 1.4 × 10^7^ GE/g, which was a higher titre in the liver compared to the one observed in other matrices, such as muscle or faeces [4,9]. However, it is also known that there can be extrahepatic replication, and it is known that the shedding of the virus is cyclical and does not occur constantly. This may explain why there is such a disparity in the positivity of the samples [39]. It is also relevant to address the fact that diagnostic methods differ in sensitivity. Even RT-PCR methods are affected by several factors and need optimization over time [36].

As for serological analysis, values found in Portugal in this study (27.6%) coincide with the range that is usually reported in Europe. Values ranging between 11.5% in Germany [40], 52.25% in Serbia [41], and 57.6% in Spain [42] were presented. However, our study shows a higher seropositivity than another Portuguese study that reports a seroprevalence of 14% in 2018, but in a smaller sample (4 seropositive in 29 samples) than ours [43]. Kozyra et al. (2020) [39] reported a correlation between serological and molecular results of HEV infection in wild boar in their study with matched samples. And, another study in Germany in 2017 developed by Anheyer-Behmenburg et al. [44,45], in Germany, in serological positive-screened wild boars, the positivity to molecular diagnosis of several tissues tested (liver, muscle, and kidney) is higher and approximately 100%. In our case, this cannot be verified by the observed low molecular prevalence in liver and faeces samples. However, seroprevalence rates in Portugal fluctuate according to the geographical location, environmental conditions, and the type of wildlife management in the studied area. When analysing in detail, it could be observed that the HEV hotspot (single positive liver sample and higher seroprevalences) also coincides with places where hunting activity is carried out with greater frequency (southeast of Central Portugal) and where other zoonoses are signalled as problematic, like tuberculosis [46,47].

As mentioned above, this disease is recognised as an emerging zoonosis with huge zoonotic potential. The dispersal of HEV raises public health concerns, especially in countries where wild animals are hunted and humans consume their meat [2,3]. In this context, evidence of HEV RNA detection in samples, like liver, muscle, and blood, raises concerns regarding the risk of transmission to humans through direct or indirect contact (e.g., hunters) [48,49] or contaminated wild boar products (e.g., undercooked meat and liver) [11]. As can be seen from the survey of 106 hunters in our study, this is a risk factor in Portugal, where a small percentage of hunters who hunt for self-consumption still consume liver, sometimes undercooked. It is also worth mentioning a high-risk practice still practised in Portugal, called hunter’s baptism (a traditional Portuguese practice after the hunter hunts a wild boar for the first time), where sometimes the liver is rubbed on hunter’s face.

Indeed, the presented HEV prevalence values underline the importance of wild boar as a reservoir of this zoonotic agent in Portugal and alert to the risk for consumers [48,50]. Several recommendations are addressed by the authors based on the literature:

(i) Cooking meat is an essential recommendation to limit the risk of HEV transmission [4,19,35,44].

(ii) As animals are directly eviscerated, hunters are thus directly exposed to blood and organs with a risk of transmission of pathogens, including HEV. It is highly recommended that hunters wear protective gloves for handling and evisceration in field conditions [35,48]. Also, particular attention should be given to wildlife managers and veterinarians [3,48].

(iii) Novice hunters in Portugal are called to perform the typical hunting baptism, which involves rubbing the freshly collected wild boar liver to the face. Thus, this behaviour can potentially pose a risk factor for HEV by direct contact with blood from infected animals, and alerts should be made [30].

(iv) Faeces can naturally contaminate wild boar meat for consumption during poor practices of evisceration and the handling of carcasses. So consequently, consuming contaminated meat from potentially infected animals could be a source of zoonotic transmission. It is necessary to alert and inform the carcasses’ handlers of this risk and to avoid the carcasses’ contamination with faeces from an intestinal rupture [16].

(v) The highly observed viral load in liver, which is the main site of virus replication, deserves attention as the liver is also used to produce regional food specialties, such as liver sausages that could be consumed raw [4].

(vi) The cuts of meat derived from animals potentially implicated in the foodborne transmission of HEV should be tested as part of the national food safety program, mainly when used for products that traditionally are consumed raw, such as dry sausages and salami-containing liver [8].

(vii) The necessity to improve official control programs for this zoonotic agent [14], as in the case of Portugal, exists a surveillance program for wildlife diseases, which HEV must be included for the wild boar population.

(viii) In the absence of specific control measures for HEV, hunters should be informed about implementing procedures during slaughtering [9,48].

In short, consuming infected wild boar meat, edible viscera or practicing inadequate measures in the hunted carcasses’ evisceration and handling could be a source of HEV zoonotic transmission.

## 5. Conclusions

Overall, this study demonstrates that HEV still circulates in wild boar in Portugal, which should be considered a crucial spreader and host of this foodborne disease. Data presented reinforce the importance of including HEV in surveillance programs for wildlife diseases to expand the existing information and assess the potential zoonotic risk of this disease, particularly for hunters and other game-related stakeholders.

HEV infection is still an underdiagnosed disease in humans and animal hosts due to the lack of diagnosis and surveillance protocols, limiting the knowledge of data about HEV occurrence. Alerts of this real zoonotic risk for consumers of this type of meat, in particular hunters, should be a priority for the authorities.

## Figures and Tables

**Table 1:** Responses of hunters’ survey (n = 106).

Question	Alternative Responses	Number (Total = 106)	Percentage (%)
“Do you usually consume the liver of the carcasses that you eviscerate and prepare?”	Never	81	76.40%
	Always	4	3.80%
	Sometimes	21	19.80%
“If so, is there any possibility of consuming the liver undercooked?” (Only for hunters that respond always or sometimes in the previous question (n = 25))	Never Always	22 0	88% 0%
	Sometimes Not applicable	3 81	2.8% 76.4%
“Have you ever seen a hunter’s baptism, the liver being rubbed on the hunter’s face?”	Never	52	49%
	Always	4	3.80%
	Sometimes	50	47.20%

## Data Availability

Data sharing not applicable.
